# Characterization of myeloperoxidase and its contribution to antimicrobial effect on extracellular traps in flounder (*Paralichthys olivaceus*)

**DOI:** 10.3389/fimmu.2023.1124813

**Published:** 2023-01-26

**Authors:** Qiujie Gan, Heng Chi, Roy Ambli Dalmo, Xianghu Meng, Xiaoqian Tang, Jing Xing, Xiuzhen Sheng, Wenbin Zhan

**Affiliations:** ^1^ Laboratory of Pathology and Immunology of Aquatic Animals, KLMME, Ocean University of China, Qingdao, China; ^2^ Laboratory for Marine Fisheries Science and Food Production Processes, Qingdao National Laboratory for Marine Science and Technology, Qingdao, China; ^3^ Norwegian College of Fishery Science, Faculty of Biosciences, Fisheries and Economics, UiT - the Arctic University of Norway, Tromsø, Norway

**Keywords:** myeloperoxidase, extracellular traps, antibiosis, immune response, fish

## Abstract

Myeloperoxidase (MPO) is a cationic leukocyte haloperoxidase and together with other proteins, they possess activities against various microorganisms and are involved in extracellular trap (ET) formation. The present work describes the gene and deduced protein sequences, and functions of MPO in flounder (*Po*MPO). The *Po*MPO possesses a 2313 bp open reading frame (ORF) that encodes a protein of 770 amino acids. The highest *PoMPO* mRNA expression levels were found in the head kidney, followed by peritoneal cells, gill, spleen, skin, muscle, and liver. *Po*MPO was expressed in MHCII^+^ and GCSFR^+^ cells which indicated that *Po*MPO mainly is expressed in flounder macrophages and granulocytes. Bacterial lipopolysaccharide-stimulated peritoneal leukocytes showed an increased protein level of *Po*MPO while it seemed that LPS also promoted the migration of MPO^+^ cells from the head kidney into the peripheral blood and peritoneal cavity. After phorbol 12-myristate 13-acetate (PMA) or bacterial stimulation, flounder leukocytes produced typical ET structures containing DNA with decoration by MPO. The ETs containing DNA and *Po*MPO effectively inhibited the proliferation of ET-trapped bacteria. Blocking *Po*MPO with antibodies decreased the enzymatic activity, which attenuated the antibacterial activity of ETs. This study pinpoints the involvement of ETs in flounder innate responses to pathogens.

## 1 Introduction

Myeloperoxidase (MPO) and eosinophil peroxidase (EPO) belong to the peroxidase superfamily and are cationic leukocyte haloperoxidases with potent microbicidal and detoxifying activities (1). MPO is a homodimeric glycoprotein localized in azurophilic granules, mainly synthesized by neutrophil and monocyte precursor cells, which constitute approximately 25% of the granule mass in these human cells (2, 3). The molecular weight of mature MPO in mammals is ~146 kDa, consisting of two ~73 kDa units joined by cysteine bridges. EPO is a peroxidase released only from eosinophils and is considered to be a specific marker for eosinophils, although it is also present in monocyte precursors. Mature EPO in mammals is a monomer of ~70 kDa, which accounts for about 40% of eosinophilic granule weight (4, 5). MPO and EPO in human are encoded by genes on chromosome 17, which share a 70% amino acid homology, suggesting that the two peroxidase genes are generated from a common ancestral gene (6, 7). MPO and EPO can both utilize H_2_O_2_ and chloride ions (Cl^-^) to generate hypochlorous acid (HOCl), which contributes to microbial killing and may contribute to protein immunogenicity (8–10). Interestingly, Marcinkiewicz et al. (11) proposed that protein modification by HOCl may act as a neutrophil-dependent molecular tagging system, indicating that neutrophils are involved in the induction stage of adaptive immunity (11).

Neutrophils are the first immune cells to be recruited to the site of inflammation and play an important role in the innate immune response by phagocytosis, degranulation, and the formation of neutrophil extracellular traps (ETs). Recently, an increasing number of granule proteins associated with neutrophil ETs have been identified as associated with ET formation, one being MPO (12, 13). In the extracellular environment, the protein composition and post-translational modifications of ETs produced under different stimuli have been studied using proteomic analysis, which revealed that methionine sulfoxide-containing aenolase (methionine replaced by methionine sulfoxide in position 93; methyl-oxidized αenolase) is the most frequent post-translational modifications across all conditions of NET formation (82%) and the majority of modified peptides (45%) are derived from MPO activity (14). However, the function of MPO and whether it contributes to antimicrobial effect on ETs are unknown in teleost.

MPO has been identified in zebrafish (*Danio rerio*), turbot (*Socophahalmus maximus*), channel catfish (*Ictalurus puncyatus*), rock bream (*Oplegnathus fasciatus*), crucian carp (*Carassius auratus gibelio*), and orange-spotted grouper (*Epinephelus coiodes*), while the EPO was only cloned in zebrafish and starry flounder (*Platichthys stellatus*) (15–22). In GeneBank, the predicted gene sequences of EPO and MPO, from whole genome sequencing of fish, are most probably not well annotated. In this study, the sequence and structure of MPO in flounder *Paralichthys olivaceus* (*Po*MPO) were determined. In addition, the contribution of *Po*MPO to antimicrobial effect in ETs is presented.

## 2 Materials and methods

### 2.1 Experimental bacteria, viruses, and animals

*Escherichia coli* (*E. coli*)*, Staphylococcus aureus* (*S. aureus*), *Edwardsiella tarda* (*E. tarda*), *Vibrio anguillarum* (*V. anguillarum*), and hirame rhabdovirus (HIRRV) strain were previously isolated from diseased flounder and stored in our laborotory (23, 24). Bacteria were cultured in Luria-Bertani (LB) medium up to an OD600 of 0.5 and harvested by centrifugation before use. Healthy flounders (15-35 cm in length) were purchased from a fish farm in Rizhao, Shandong Province, China, and were acclimatized in recirculating seawater at 21 °C for two weeks. During the acclimatization, fish were fed daily with commercial dry food pellets. BALB/c mice were obtained from Qingdao Animal Experimental Center of Shandong Province, China, and used for antibody production. The protocols for animal care and handling were approved by the Animal Care and Use Committee of Ocean University of China (Permit Number: 20180101). All possible efforts were dedicated to minimizing suffering.

### 2.2 Challenge and sampling

For infection, flounders were randomly divided into four groups (30 fish per group). Fish in each group were intraperitoneally (i.p.) injected with *V. anguillarum*, *S. aureus* (1 × 10^6^ CFU per individual), or HIRRV (1 × 10^7^ TCID_50_ per individual). The fish in the control group was injected with the same volume of PBS. At 0, 12, 24, 48, and 96 h post-injection, the head kidney and spleen of five fish from each group was sampled for expression profile analysis. All samples were immediately frozen in liquid nitrogen and then stored at -80 °C until RNA isolation.

### 2.3 Sequence analysis of *Po*MPO

Total RNA was extracted from tissues or cells of flounder by a Trizol method reported previously (25). The cDNA strand was synthesized using a Hiscript III RT SuperMix kit for PCR (Vazyme, Nanjing, China). The full-length open reading frame (ORF) of the *Po*MPO gene was amplified using primers *Po*MPO-OF/*Po*MPO-OR (**Table 1**). The gene sequences and location in the genome of MPO were analyzed in the NCBI database. The SMART, IEDB-AR, and ExPASy Molecular Biology Server were used to analyze the protein structure, antigenic epitopes, and physicochemical properties. MEGA 5.0 software was used to construct and analyze a phylogenetic tree using the neighbor-joining method with 1,000 bootstrap trials (26, 27).

**Table 1:** The primers used in this study.

Primer name	Sequence (5’→3’)
*Po*MPO-OF	ATGTTTCTCTCTGTCCTGCTCG
*Po*MPO-OR	CTACTCCACCTCGTTGTCCTG
*Po*MPO-F (EcoRI)	CGGAATTCAAGTATCGCACAATCACCAGC
*Po*MPO-R (XhoI)	CCCTCGAGAAGGAAACACGGCACCTC
qPCR-F	AGATCTGTCCCGATGAACGC
qPCR-R	TTACAGCTATCACCCGAGCC
18S-F	GGTCTGTGATGCCCTTAGATGTC
18S-R	AGTGGGGTTCAGCGGGTTAC
T7F	TAATACGACTCACTATAGGG
T7R	TGCTAGTTATTGCTCAGCGG

Restriction site with an underline.

### 2.4 Preparation of anti-r*Po*MPO antibody

The sequence (427-1155 bp) with strong antigenicity of *Po*MPO was amplified using primers *Po*MPO-F/*Po*MPO-R (**Table 1**) and cloned into the pET32a vector to construct recombinant pET32a-*Po*MPO expression plasmid. *E. coli* BL 21 (TransGen Biotech, Beijing, China) was transformed by pET32a-*Po*MPO and cultured with LB medium at 37 °C. After growth to log phase (OD 600 = 0.6-0.8), the bacteria were induced with IPTG (Isopropyl β-D-Thiogalactoside, 250 µg mL^-1^) and incubated overnight at 16 °C. Then the recombinant protein of *Po*MPO (r*Po*MPO) was obtained by affinity chromatography using His Trap™ HP Ni-Agarose (GE healthcare China, Beijing, China) (28).

The concentration of r*Po*MPO was determined to be 1 mg mL^-1^ by using the Bradford kit (P0006C, Beyotime, Shanghai, China). BALB/c mice were immunized with 100 µg r*Po*MPO according to previously reported methods (29). After two boosters with a mixture of r*Po*MPO and incomplete Freund’s adjuvant (Sigma, St. Louis, MO, USA), the mouse serum was collected and purified the antibodies with protein G-agarose column (Pierce/Thermo Scientific, Waltham, Massachusetts, USA). The anti-r*Po*MPO Abs titer was tested by enzyme-linked immunosorbent assay, and then the specificity was analyzed using Western blotting and Mass spectrometry analysis (Sangon Biotech, Shanghai, China).

### 2.5 Isolation of leukocytes from the head kidney, peripheral blood and peritoneal cavity

The leukocytes in the head kidney, peripheral blood and peritoneal cavity of flounder (30-35 cm) were isolated according to the method described previously (30). In brief, the peripheral blood was drawn from the caudal vein and diluted in solution (65% RPMI-1640 containing 20 IU mL^−1^ heparin, 0.1% w/v NaN3, and 1% w/v BSA). The head kidney was passed through a nylon falcon filter while grinding and adding L-15 medium to form cell suspension. Then the leukocytes were isolated by percoll (GE Healthcare, Uppsala, Sweden) on a discontinuous density gradient (1.020-1.070 g mL^-1^). Finally, the interfacial cells were collected and resuspended in L-15 medium at a density of 1×10^6^ cells mL^-1^. For peritoneal cell isolation, L-15 medium was intraperitoneally (i.p.) injected into flounder and then gently massaged for 5 min. The peritoneal fluid was collected along the edge of the peritoneal cavity by a syringe and centrifugation at 450 × *g* for 10 min. The cell pellet was resuspended and adjusted to a concentration of 1×10^6^ cells mL^-1^.

### 2.6 Flow cytometry

The peripheral blood leukocytes (PBLs), head kidney leukocytes (HKLs) and peritoneal cells (PerCs) were obtained from healthy or LPS (1mg mL^-1^) -stimulated flounder (i.p injection of LPS). After being adjusted to 5 × 10^6^ cells mL^-1^ in PBS, the potentially unspecific staining was reduced by adding 5% BSA and incubated with antibodies (rabbit anti-*Po*MHCII or anti-*Po*GCSFR Abs) for 1 hour at room temperature (BD Cytofix/Cytoperm kit; BD Biosciences) (31, 32). Afterwards, the cells were incubated with Abs [mixture of mouse anti-r*Po*MPO and rabbit anti-Zap-70 (Cell Signal Technology, Danvers, Massachusetts, USA)] for 1 hour at room temperature (33). Mouse anti-Trx Abs and rabbit anti-Trx Abs were used as negative controls. The cells were subsequently incubated with Alexa Fluor 649-conjugated goat anti-mouse IgG or 488-conjugated goat anti-rabbit IgG Abs for 45 min at 37°C in the dark. Flow cytometry was performed using FACSCalibur (BD Biosciences) flow cytometers with acquisition enabled by CellQuest Pro software.

### 2.7 Immunofluorescence staining

The leukocytes (1×10^6^ cells mL^-1^) were seeded onto circular coverslips (14 mm, Solarbio, Beijing, China) pretreated with 0.01% polylysine (Sigma, St. Louis, MO, USA), and placed on 24-well cell culture plates (Corning Costar, Cambridge, MA, USA) to settle for 1 h. The cells were fixed with 4% paraformaldehyde (Solarbio, Beijing, China) and permeabilized by 0.25% TritonX-100 in 5% BSA. Then cells were incubated with mouse anti-r*Po*MPO Abs, or the mixture of antibodies - mouse anti-r*Po*MPO Abs together with rabbit anti-Zap-70 Abs, rabbit anti-MHCII Abs, or rabbit anti- GCSFR Abs overnight at 4 °C, followed by incubation with Alexa Fluor 649-conjugated goat anti-mouse IgG or 488-conjugated goat anti-rabbit IgG secondary Abs for 1 h at 37 °C. After washing, the cells were counterstained with 2-(4-Amidinophenyl)-6-indolecarbamidine dihydrochloride (DAPI, Thermo Scientific, Waltham, Massachusetts, USA) and visualized by fluorescence microscopy (ZEISS, Oberkochen, Germany). The mouse anti-Trx and rabbit anti-Trx Abs were used as negative controls.

### 2.8 Western blotting

According to the user-instruction of the RIPA Lysis Buffer Kit (Beyotime, Shanghai, China), 100 µL of lysis buffer was added to 1 × 10^6^ peritoneal cells pellet or 10 mg tissue. After ultrasonication for 20 min, the lysate was centrifuged at 12000 × *g* for 30 min, and then the protein concentration in the supernatant was adjusted to 1mg mL^-1^ by using the BCA kit (EpiZyme, Shanghai, China). Twenty microliters of the cell or tissue (head kidney, spleen, gill, skin, muscle, and liver) lysates went through SDS-PAGE and transferred onto polyvinylidene fluoride (PVDF) membrane (Merck Millipore, Darmstadt, Germany). Then the membranes were blocked with 5% BSA and incubated with anti-r*Po*MPO, anti-Trx, or anti-GAPDH Abs (latter ab: Abclonal, Wuhan, China). After washing three times with PBST, the membranes were incubated with goat anti-mouse IgG-HRP or goat anti-rabbit IgG-HRP (Sigma, St. Louis, Mo, USA) diluted with 5% BSA at 37 °C for 45 min. Finally, the bands were detected with ECL Enhanced Kit (Abclonal, Wuhan, China) in a chemiluminescence detection instrument (Vilber, Ile-de-France, France). The semi-quantification of protein bands from homogenized PerCs priority subjected to LPS (100 µg per individual (N = 3)), Sigma, St. Louis, Mo, USA) at different time points was analyzed using Image J software (34).

### 2.9 Real-time quantitative PCR

The Real-time quantitative PCR (qPCR) reaction system contained 10 µL 2 × Universal SYBR Green Fast qPCR Mix (Abclonal, Wuhan, China), 10 ng sample DNA, 0.4 µL detection primer qPCR-F/qPCR-R or 18S-F/18S-R (**Table 1**) and sterile distilled water for a final volume of 20 µL. The qPCR amplification reaction program was set up as follows: 95 °C for 3 min, followed by 40 cycles at 95 °C for 5 s, 60 °C for 30 s. The relative transcriptional levels of *Po*MPO were calculated by normalizing the abundance of 18S as an internal control. The relative expression of *Po*MPO was analyzed using the 2^-ΔΔCt^ method based on the Ct (cycle threshold) values generated by the software automatically.

### 2.10 Visualization of ETs by scanning electron microscopy

The leukocytes (1×10^6^ cells mL^-1^) from the head kidney on circular coverslips were stimulated with PMA (100 ng mL^-1^), *E. coli*, *E.tarda*, or *S. aureus* (1×10^8^ CFU mL^-1^) for 3 h respectively, and then fixed with 2.5% glutaraldehyde (Hushi, Shanghai, China) at 4 °C in the dark for 2 h. Next, the samples were dehydrated by adding ethanol (Hushi, Shanghai, China) by a graded series (30%, 50%, 70%, 80%, 90%, 100%) for 15 min of each at room temperature. Samples were treated with isoamyl acetate (Sigma, St. Louis, Mo, USA) for 20 min, subjected to critical point drying for 3 h (Hitachi-HCP, Hitachi, Tokyo, Japan), coated with gold (MC1000, Hitachi, Tokyo, Japan) and viewed under SEM (S-3400N, Hitachi, Tokyo, Japan).

### 2.11 Measurement of fluorescence intensity changes of ETs *in vitro*

The measurement of fluorescence intensity changes of ETs was examined as the methods reported previously (35). Briefly, to quantify ETs, HKLs were suspended in phenol red-free L-15 medium and seeded in black 96-well plates (Cayman Chemical, Ann Arbor, MI, USA). The cells were stimulated with PMA (100 ng mL^-1^), live *E. coli*, *E. tarda*, or *S. aureus* (1 × 10^5^ CFU mL^-1^) for 1, 2, and 3 h, while the containing cytochalasin D in each group (20 µg mL^−1^, Absin, Shanghai, China) functioned as control. After stimulation, Sytox Green (5 µM, Thermo Scientific, Waltham, Massachusetts, USA) was added to the wells to dye the extracellular DNA. Fluorescence was then quantified as relative fluorescence units (RFU) at 485 nm excitation and 520 nm emission using a fluorescence spectrophotometer (Infinite M26000, FLUOstar Omega, Germany).

### 2.12 Detection of MPO activity

To determine whether anti-r*Po*MPO Abs inhibits MPO activity, the supernatant of HKLs was incubated alone, or with anti-r*Po*MPO Abs (1:1000) for 45 min. The activity of MPO was measured by the MPO colorimetric activity assay kit (Nanjing Jiancheng Co., Ltd., China) according to the manufacturer’s instructions. Finally, the absorbance was measured at 460 nm to evaluate the MPO activity.

### 2.13 Survival of ETs-trapped bacteria

The survival of ETs-trapped bacteria was examined as the methods reported previously (35–37). Briefly, HKLs (1×10^6^ cells mL^-1^) were suspended in L-15 medium and seeded in 96-well plates to adhere for 60 min at 22 °C. The cells were stimulated with PMA for 2 h to induce the ET formation, while control cells were added with the same volume of L-15 medium. Then, L-15 medium containing cytochalasin D (20 µg mL^−1^) was added to the wells of the plates. In addition, some wells were added anti-r*Po*MPO Abs (1:1000). After treatment for 45 min, 2,000 CFU *E. coli*, *E. tarda*, or *S. aureus* in 100 µL L15 medium were added to wells, respectively. After incubation at 0, 2, 4, and 8 h, the content from each well were taken out, diluted, and plated on LB agar plates. Finally, after incubation at 30°C for 24 h, the colonies appearing on the plates were counted.

### 2.14 Statistical analysis

Student’s t-test or the one-way analysis of variance (ANOVA) and Duncan’s multiple comparisons were performed by using Statistical Product and Service Solution (SPSS) software (Version 19.0; SPSS, IBM, BY, USA). In all cases, *P* < 0.05 was considered significant.

## 3 Results

### 3.1 Sequence analysis of *Po*MPO

**Figure 1:**
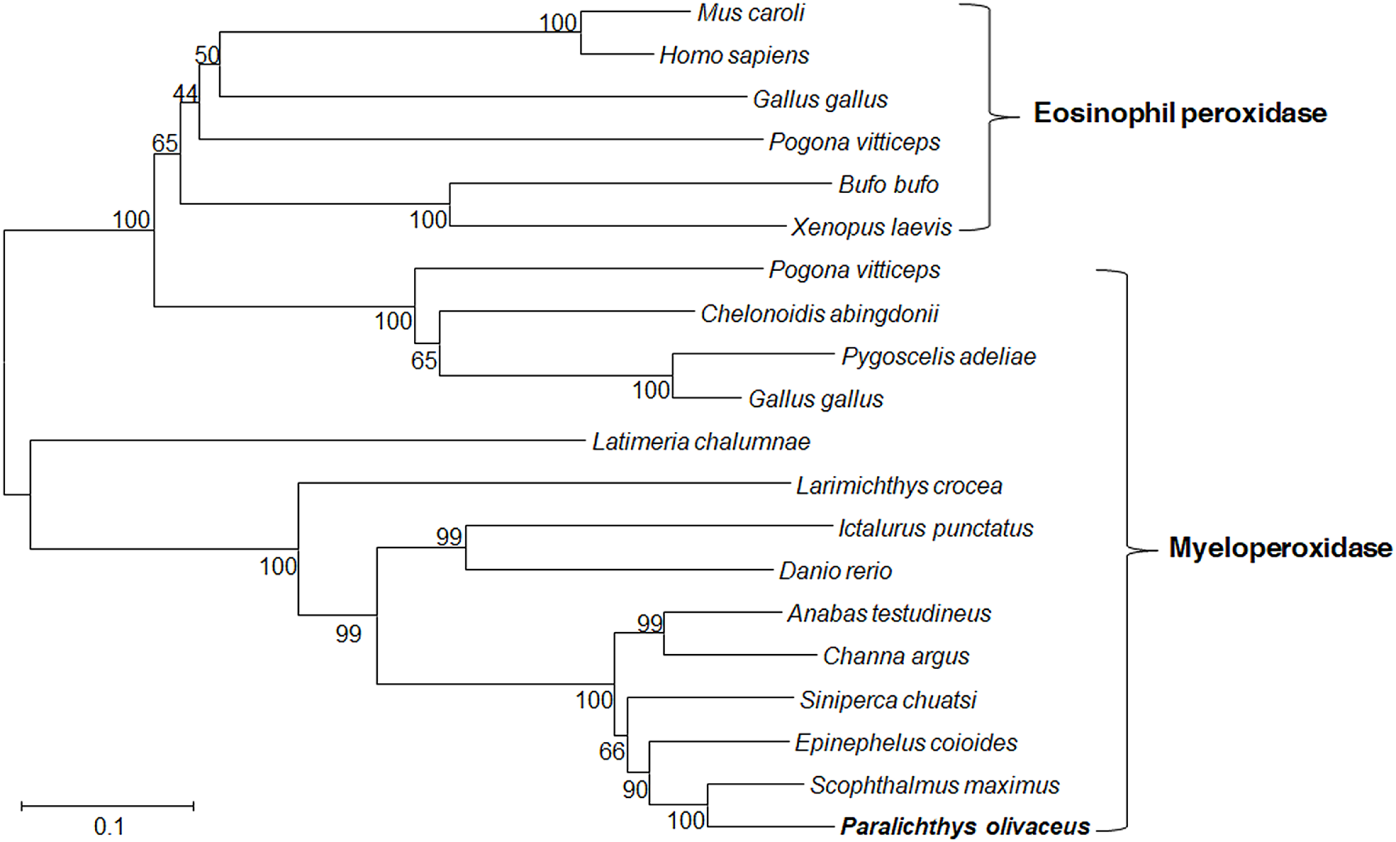
Phylogenetic tree showed the relationship between flounder MPO/EPO and other vertebrate amino acid MPO/EPO sequences. Numbers in each branch indicate the percentage bootstrap values from 1000 replicates. The accession numbers of MPO and EPO amino acid sequences are as follows, MPO: *Paralichthys olivaceus*, XP_019938121.1; *Siniperca chuatsi*, ABC72122.1; *Danio rerio*, AAK83239.1; *Pogona vitticeps*, XP_020659555.1; *Gallus gallus*, XP_015151399.1; *Epinephelus coioides*, APM83155.1; *Channa argus*, QCY41338.1; *Pygoscelis adeliae*, KFW66803.1 (partial); *Anabas testudineus*, XP_026195736.1; *Larimichthys crocea*, KAE8292175.1; *Latimeria chalumnae*, XP_005992326.1; *Chelonoidis abingdonii*, XP_032625834.1; *Ictalurus punctatus*, ACV69995.1; *Scophthalmus maximus*, XP_035464009.1. EPO: *Gallus gallus*, XP_015151415.1; *Pogona vitticeps*, XP_020659588.1; *Homo sapiens*, AAA58458.1; *Xenopus laevis*, NP_001081848.1; *Bufo bufo*, XP_040279578.1; *Mus caroli*, XP_021033325.1.

**Figure 2:**
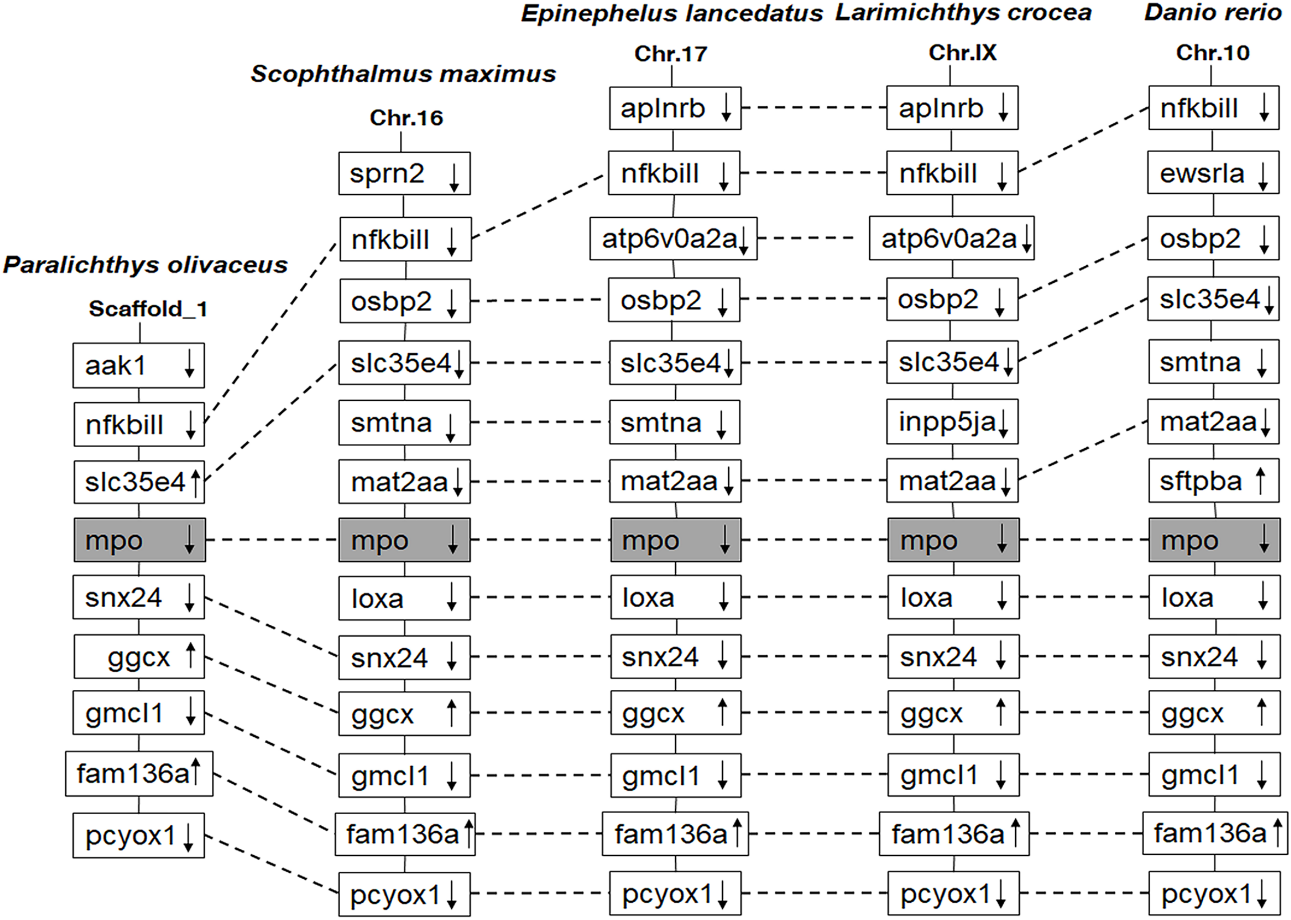
Comparison of the location of MPO genes in different fish species. The chromosomes and scaffold were identified in NCBI. Boxes represent the deduced genes. Arrows indicate the deduced orientation of gene transcription. Dashed lines connecting boxes suggest a homologous relationship.

The *Po*MPO cDNA sequence (GenBank accession no. XM_020082562.1) consisted of an ORF of 2313 bp encoding a protein of 770 amino acids (**Supplementary Figure 1A**). The putative protein had a molecular mass of 86.928 kDa and an estimated pI of 9.21. Secondary structure analysis exhibited that *Po*MPO comprised signal peptides and a peroxidase domain (**Supplementary Figure 1B**). The multiple amino acid sequence alignment results show several conserved domains such as the signal peptide (1-18 aa), propeptide (19-104 aa), light (105-248 aa), and heavy chains (249-770 aa). Other important sites for regulation of MPO activity, including propeptide cleavage site (98-104 aa), haem cavity, and Ca^2+^-binding motif (312-319 aa), are also present in *Po*MPO. The similarity of MPO between flounder and other animals ranged from 62.4% to 87.4%. (**Supplementary Figure 2**). A phylogenetic tree was built with MPO and EPO from fish, frog, chicken, and mammals showed that the *Po*MPO formed a separate clade with MPO sequences of teleost and its closest relationship is confirmed to be with the turbot (**Figure 1**). Furthermore, the motifs of at least ten genes at adjacent positions of the MPO in five teleost species were analyzed to obtain more information about the origin of the *Po*MPO gene. The analysis revealed a conserved set of genes (nfkbil1, slc35e4, snx24, ggcx, gmcl1, fam136a, pcyox1) in the vicinity of the MPO gene in teleost (**Figure 2**).

### 3.2 Production of mouse anti-MPO antibody

The SDS-PAGE analysis showed that r*Po*MPO-Trx (46 kDa) was successfully expressed in *E. coli* BL 21. The recombinant proteins of *Po*MPO-Trx with high purity were obtained and used for the production of antibodies (**Supplementary Figure 3A**). Western blotting verified that the anti-r*Po*MPO Abs could specifically recognize r*Po*MPO (46 kDa) and endogenous *Po*MPO (75 kDa) (**Supplementary Figure 3B**). The mass spectrometry results showed that the protein sequence of this 75 kDa protein similar to the theoretical *Po*MPO - with 46% sequence identity (**Supplementary Figures 3C, D**). From the Western blot experiment, the anti-*Po*MPO Abs specifically recognized *Po*MPO - this polyclonal antibody can be used to target *Po*MPO in functional studies.

### 3.3 The profile of *Po*MPO expression in tissues and peritoneal cells

**Figure 3:**
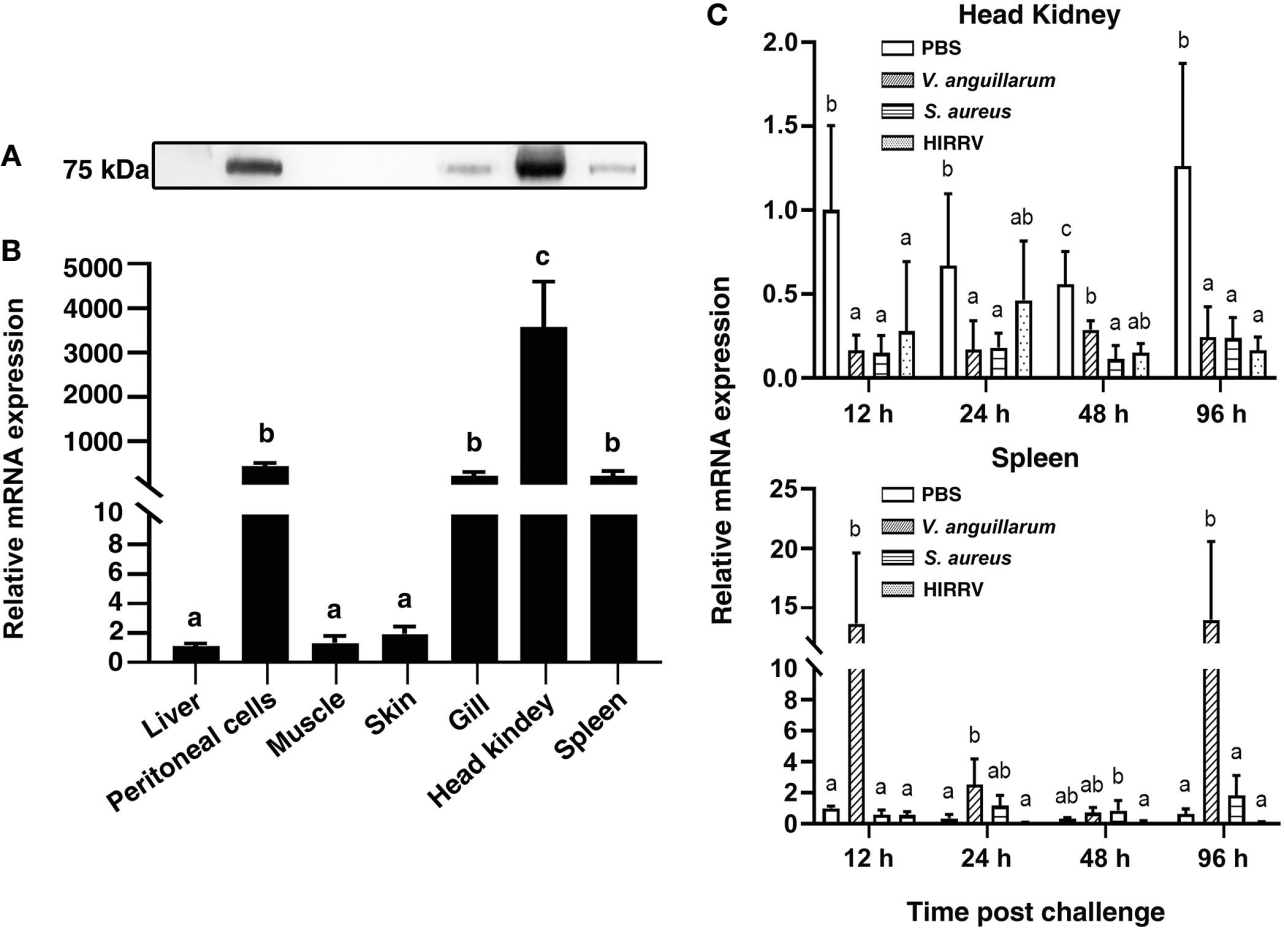
The expression of *Po*MPO was determined by Western blotting **(A)** and quantitative real-time PCR **(B)** in different tissues. **(C)** Relative gene expression of *Po*MPO in the head kidney at different time points after *V. anguillarum*, *S. aureus*, or HIRRV infection. The results were calculated using relative expression method with 18S as the housekeeping gene. Different letters above the bar represent the statistical significance (*p* < 0.05) compared to each other at the same time point, and vertical bars represented the mean ± SD, n = 5.

The *Po*MPO gene was widely expressed in all detected tissues. The highest expression levels of *Po*MPO mRNA were found in the head kidney, followed by peritoneal cells, gill, spleen, skin, muscle, and liver (**Figure 3A**). In agreement with the mRNA expression pattern, *Po*MPO protein was found mainly in the head kidney, peritoneal cells, gill, and spleen. In contrast, no clear bands were detected in skin, muscle, and liver (**Figure 3B**). To assess the involvement of *Po*MPO in the response of *V. anguillarum*, *S. aureus*, and HIRRV infection, its transcriptional level was examined in the head kidney and spleen at different time points. The expression of *Po*MPO mRNA in samples taken from infected fish was significantly lower compared with the expression in samples taken from control fish at all time points examined (*P* < 0.05) (**Figure 3C**). In the spleen, *Po*MPO expression was significantly upregulated after infection with *V. anguillarum* and peaked at 96 h (*P* < 0.05). However, after infection with *S. aureus* or HIRRV, the expression of *Po*MPO was not significantly different from the PBS group in the spleen (**Figure 3C**). For further verification, quantitative analysis of HKLs by western blot showed that the content of *Po*MPO protein was decreased after stimulated by *V. anguillarum* for 12 h. However, the content of *Po*MPO protein in spleen showed no significant change compared to the control group (**Supplementary Figure 4B**).

### 3.4 Possible migration of MPO^+^ cells from head kidney to blood and the peritoneal cavity

**Figure 4:**
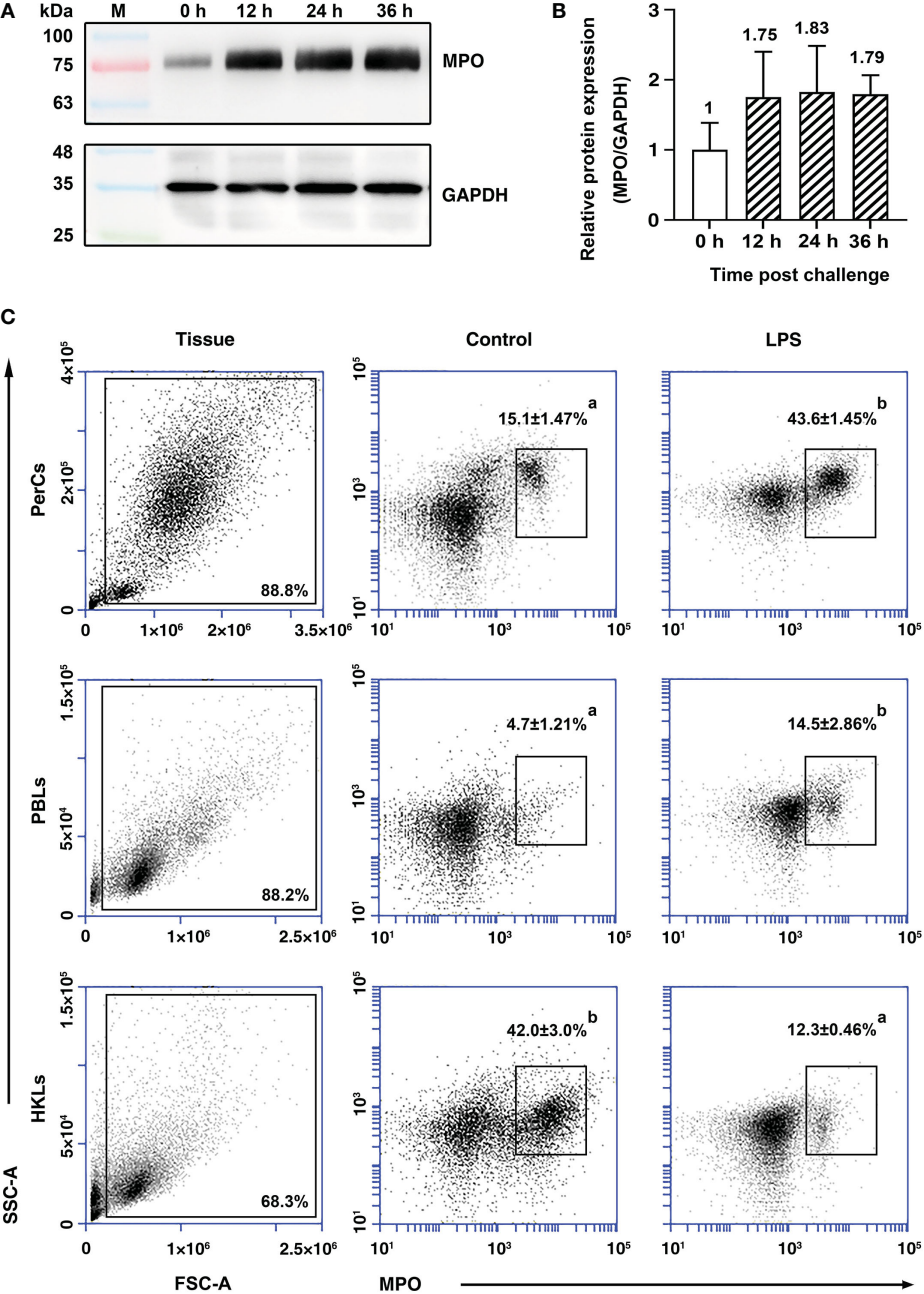
The expression of *Po*MPO in leukocytes at different time points upon LPS stimulation. **(A)** Cell lysates were harvested and subjected to Western blotting analysis using anti-r*Po*MPO and anti-GAPDH Abs and the semi‐quantification of *Po*MPO was shown **(B)**. **(C)** Flow cytometric analysis of MPO^+^ peritoneal leukocytes in the gate of FSC area (FSC-A)/SSC area (SSC-A) of flounder. Data are representative from three independent experiments, and vertical bars represent the mean ± SD. Different letters represent the statistical significance (*p* < 0.05) compared to each other at the same tissue.

After stimulation with LPS *in vivo*, the content of *Po*MPO protein in PerCs was found to be elevated at all sampling time points compared with 0 h (**Figure 4A**). The analysis showed that the content of *Po*MPO was increased approx two-fold after LPS stimulation (*p* = 0.2350) (**Figure 4B**). By immunofluorescence staining and analysis, the number of MPO^+^ cells increased in the peritoneal cavity and in the peripheral blood following LPS stimulation, while the number was reduced in the head kidney after LPS stimulation (**Supplementary Figure 4A**). Flow cytometric analysis showed that the percentage of MPO^+^ cells increased from 15.1 ± 1.47% to 43.6 ± 1.45% in the peritoneal cavity and from 4.7 ± 1.21% to 14.5 ± 2.86% in peripheral blood after LPS stimulation. The percentage of head kidney MPO^+^ cells was, however, reduced (from 42.0 ± 3.0% to 12.3 ± 0.46%) (**Figure 4C**) following i.p. injection of LPS.

### 3.5 Identification of *Po*MPO^+^ cell types

**Figure 5:**
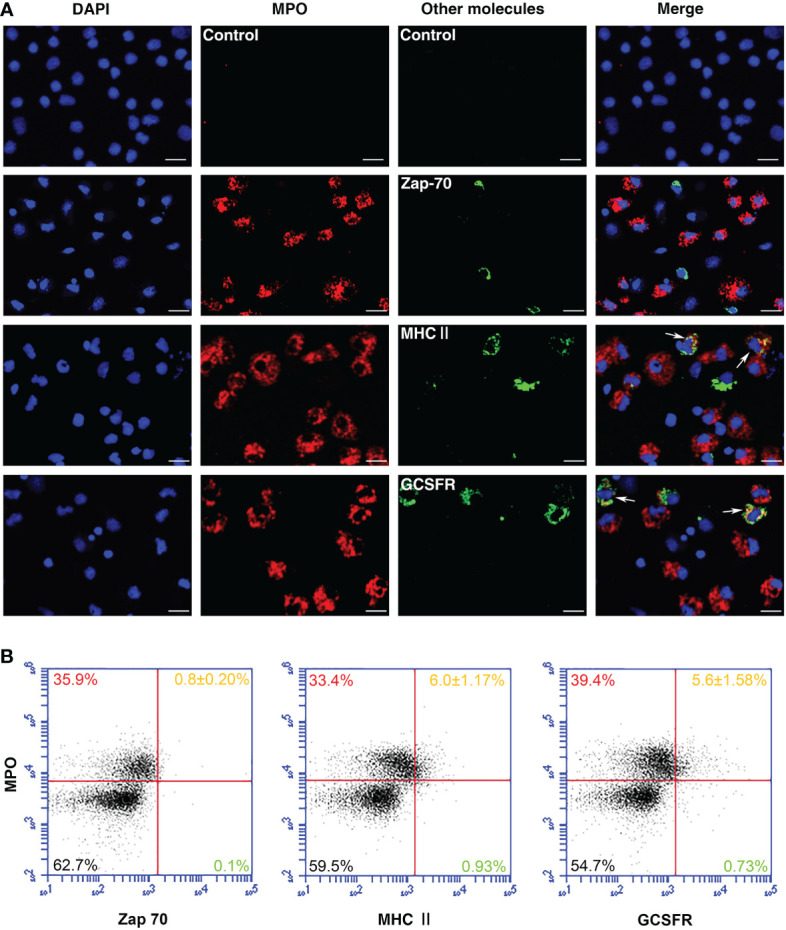
Identification of MPO^+^ cells in flounder. **(A)** Peritoneal cells were double-stained using anti-r*Po*MPO Abs (red) and anti-Zap-70, anti-MHCII, or anti-GCSFR Abs (green), respectively. The blue color showed the DAPI dye nuclei. Arrows indicated double-positive cells. Bar = 10 µm. **(B)** Peritoneal cells were double-stained and analyzed by flow cytometry. Each figure is representative from three analysis (mean ± SD, n = 5).

For the identification of *Po*MPO^+^ cell types, double immunofluorescence staining of PerCs was performed. The results revealed also the presence of MHCII and GCSFR proteins in *Po*MPO^+^ cells. However, by immunocytochemical analysis, the cells did not stain dually positive using both anti-*Po*MPO and anti-Zap-70. No fluorescence was observed in the negative controls (**Figure 5A**). The flow cytometric analysis of PerCs showed that the percentage of MPO^+^/MHCII^+^ cells and MPO^+^/GCSFR^+^ cells were 6.0 ± 1.17% and 5.6 ± 1.58%, respectively. The proportion of MPO^+^/Zap70^+^ cells was very low (**Figure 5B**).

### 3.6 Localization of *Po*MPO on ETs

**Figure 6:**
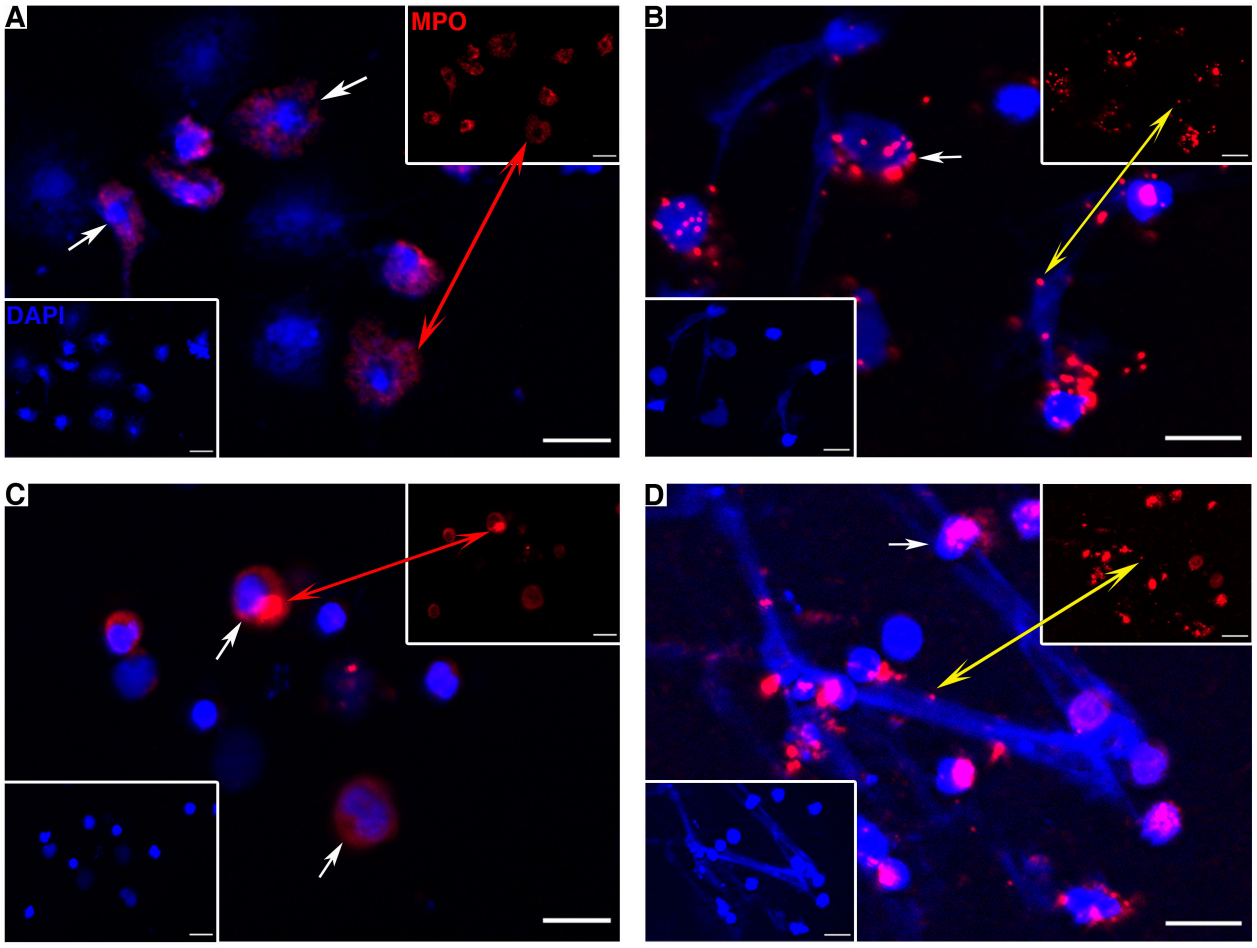
*Po*MPO positive cells (continuous arrows or red double arrow) and MPO protein on ETs (yellow double arrow) in peritoneal cells **(A, B)** and head kidney leukocytes **(C, D)**. **(A, C)** show the control cells without stimulation; **(B, D)** show PMA-induced ET formation. Blue indicates the nuclei and DNA fibers stained by DAPI. Bar = 10 µm.

Leukocytes from the peritoneal cavity and head kidney were isolated and stimulated by PMA to induce the formation of extracellular DNA scaffold containing *Po*MPO. As shown in **Figure 6**, the extracellular DNA released by MPO^+^ cells from the peritoneal cavity (**Figures 6A, B**) and head kidney (**Figures 6C, D**) were stained blue by DAPI. In addition, *Po*MPO proteins were also detected in the ETs (**Figures 6B, D**). The control group used mouse anti-Trx Abs that were not observed positive cells (data not shown).

### 3.7 ET-formation and entrapment of bacteria

**Figure 7:**
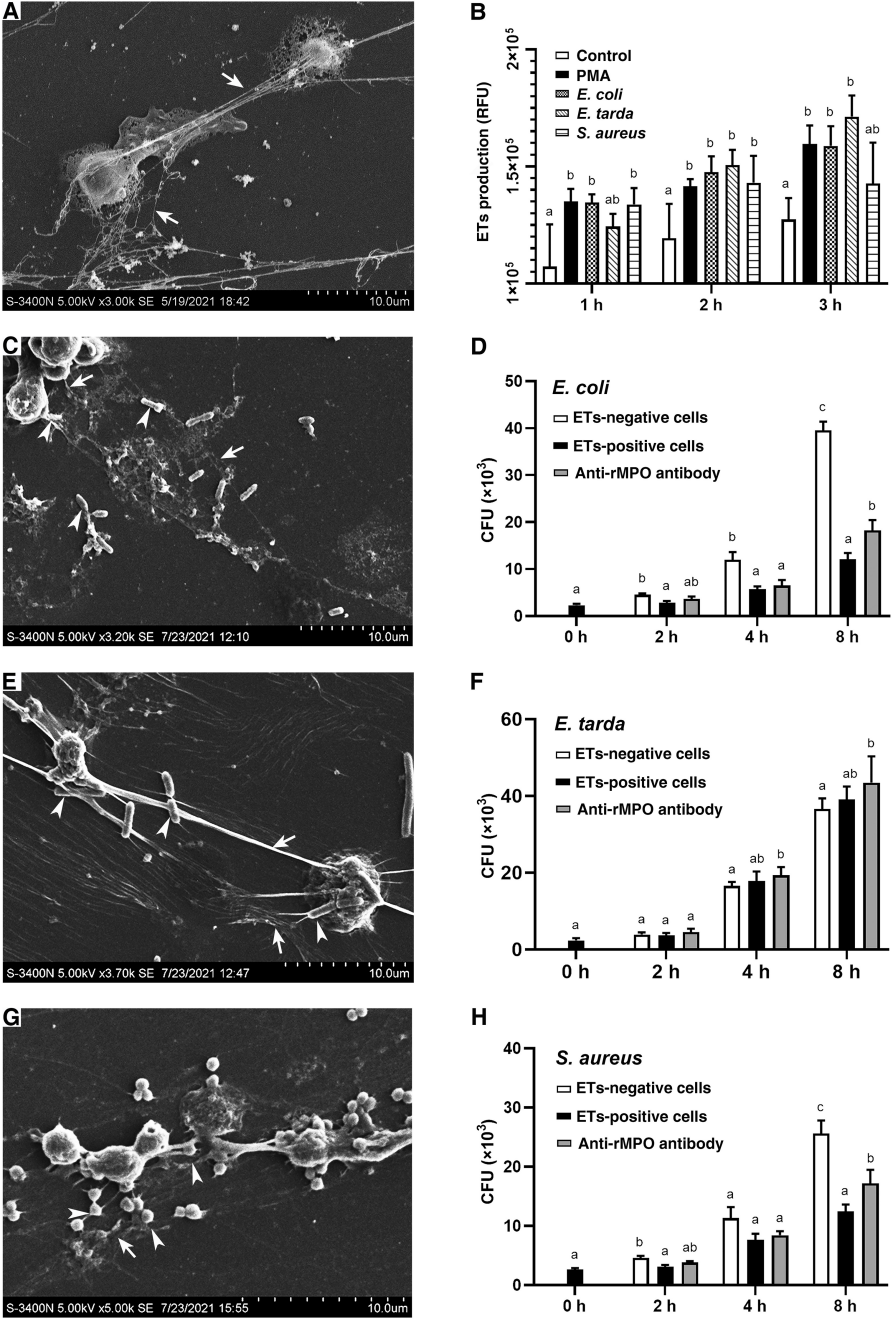
SEM analysis of the entrapment of bacteria by ETs and the proliferation of the entrapped bacteria. **(A)** PMA-induced production of ETs. **(B)** The quantitative analysis of the formation of ETs by leukocytes in response to PMA, *E. coli*, *E. tarda*, and *S. aureus* for 3 hours. **(C, E, G)** show *E. coli, E. tarda*, and *S. aureus* induced the ET formation (arrows with tail) and entrapped bacteria (arrows without tail), respectively. **(D, F, H)** show the effect of *Po*MPO on the antimicrobial activity of ETs. ETs-producing cells were incubated with *E. coli*, *E. tarda, or S. aureus* in the presence or absence of anti-*Po*MPO Abs. Bacterial survival was determined at various time points. Different letters above the bar represent the statistical significance compared to each other at the same time point, and vertical bars represented the mean ± SD, n = 5 (*P*< 0.05).

To examine whether PMA and bacteria could induce the formation of ETs in flounder, the HKLs were incubated with PMA, *E. coli*, *E. tarda*, or *S. aureus*. The quantity of ET-formation (fluorescence) from stimulated cells was significantly higher than from control cells at all examined time points after stimulation (*P* < 0.05) (**Figure 7B**). To identify the morphology of ET-formation and entrapment of bacteria, ultrastructural studies using SEM were performed and revealed that netlike structures were formed as long fibers alone or coalesced into bundles with some small particles attached. In addition, the entrapment of coccoid- or rod-shaped bacteria on the extracellular fibers was clearly observed (**Figures 7A, C, E, G**).

### 3.8 Effect of inhibiting *Po*MPO on the antimicrobial activity of ETs

Under *in vitro* conditions, the neutralization of anti-r*Po*MPO Abs was determined. The results showed that the MPO´s enzymatic activity was significantly reduced after adding anti-r*Po*MPO Abs (*p* = 0.0167) (**Supplementary Figure 5**). To examine whether *Po*MPO had any effect on the viability of the trapped bacteria, ETs-positive cells, ETs-negative cells, ETs-positive cells incubated with anti-r*Po*MPO Abs were incubated with *E. coli*, *E. tarda*, or *S. aureus* for 0 h, 2 h, 4 h, or 8 h. Then, viable bacteria were quantified. The recoveries of *E. coli* and *S. aureus* from the ETs-positive cells groups were significantly lower than ETs-negative cells at 2 h and 8 h of incubation. The number of recovered live *E. coli* and *S. aureus* in ETs-positive cells treated with anti-r*Po*MPO Abs were significantly higher compared to the ETs-positive cells at 8 h. (**Figures 7D, H**). However, for *E. tarda*, bacterial recoveries were not significantly different among the three groups at the same examined time points (**Figure 7F**).

## 4 Discussion

MPO has been mainly found at the sites of neutrophil accumulation and is reported to serve an important role in host defense, inflammation, and neutrophil function (38). Although the sequence of MPO has been identified in human, bovine, cat, Chinese alligator, frog, and different species of fish (39–43), the function and evolution of MPO in low vertebrates is still unclear. The molecular mass of mammals’ native MPO has been reported to be approximately 150 kDa and contains two monomers exhibiting a heavy (55-64 kDa) and a light (10-15 kDa) subunit in each monomer (4). In turbot, the size and structure of mature MPO are similar to mammals (16). In our study, the full-length monomeric *Po*MPO was found to have a theoretical molecular weight of 86.928 kDa, contained a signal peptide, a leading peptide, a light- and heavy chains. However, because some parts of the polypeptide are to be eliminated during maturation, the size of occurring *Po*MPO is about 75 kDa. The *Po*MPO also has one Ca^2+^ binding site, which would increase neutrophil MPO activity. The phylogenetic tree showed the *Po*MPO to be clustered with teleost homologues, quite distant from mammalian orthologs. The *Po*MPO loci shared orthologous genes in other teleost, suggesting that MPO is highly conserved during bony fish evolution.

*V. anguillarum*, *S. aureus*, and HIRRV are common pathogens with a wide range of hosts including a large number of fish. *V. anguillarum* can move across the intestinal epithelium by endocytosis, followed by the release of the bacteria that enter the blood, resulting in septicemia or the infection of various internal organs, such as the liver, spleen, and kidney (44). Abscesses and septicemia caused by *S. aureus* can also be observed in zebrafish *via* the injection of bacteria in the blood (45). HIRRV infection is characterized by a pronounced viremia, and the high viral load is typically detected in immune-related organs and the circulatory system (46). It has been shown in rats, that MPO expression in circulating neutrophils was significantly decreased after *S*. *aureus* infection (47). For comparison, the level of MPO mRNA in immune organs has been found to decrease significantly after bacterial challenge – as reported in crucian carp, rock bream, and orange-spotted grouper (18–20). Here, we found that *Po*MPO transcripts were widely expressed in the tissues of healthy flounder, and the highest expression was found in the head kidney. The expression of *Po*MPO mRNA in the head kidney was significantly reduced after i.p. injection with *V. anguillarum*, *S. aureus*, and HIRRV. These down-regulations may reflect that pathogens injected by i.p. interacted with resident peritoneal cells and susceptible immune tissue in the peritoneal cavity, which led to more MPO^+^ cells being recruited.

In mammals, a large of neutrophils spend their lives in the bone marrow, as a bone marrow reserve. Upon signaling, they will respond and mobilize into circulating neutrophils (48, 49). Neutrophils represent the most prominent leukocyte type in human peripheral blood (40%–70% of WBCs), but only constitute less than 5% of circulating leukocytes in bony fish (50). When flounder were i.p. injection with LPS, the *Po*MPO protein level in the peritoneal cavity cells was significantly increased, and the percentage of MPO^+^ cells was also increased. Our results are consistent with findings from mammalian species. In our study, the head kidney of flounder was found to contain significant neutrophilic populations, where the percentage of kidney MPO^+^ cells decreased to nearly 10% after LPS was injected intraperitoneally. The numbers of circulating MPO^+^ cells and PerCs MPO^+^ cells were increased about three-fold over basal levels. This suggests that the head kidney has a pool of MPO^+^ cells - similar to that of mammalian bone marrow. Upon mobilization caused by local inflammation, MPO^+^ cells may likely migrate from the head kidney to e.g., the peritoneal cavity.

In mammals, MPO is usually used as an intracellular marker of neutrophil accumulation in tissues and a marker of neutrophil activity in plasma. Although the expression of MPO in monocytes has been reported, it still is a matter of debate. Chi et al., 2017 reported that a potassium iodide and oxidized pyronine Y (KI-PyY) stained MPO^+^ cells in turbot exhibited characteristics of neutrophil morphology (51). Other markers used to differentiate different types of cells are: Zap-70 (Zeta-chain-associated protein kinase 70) a phylogenetically conserved protein used to visualize the presence of both T and NK lymphocytes (33). MHCII is a heterodimer preferentially expressed in macrophages, B cells, and dendritic cells (DCs), while slightly expressed in monocytes and immature DCs (31, 52). GCSFR is a class I cytokine receptor superfamily member and expressed on hematopoietic cells such as monocytes, as well as some non-hematopoietic cells, neutrophils, and their precursors (32, 53). In this study using immunocytochemistry, the *Po*MPO, and Zap-70 proteins were not found in the same cell suggesting that *Po*MPO^+^ cells are different from Zap-70 positive cells, while some *Po*MPO positive cells expressed MHCII or GCSFR proteins. Consequently, the results indicated that *Po*MPO mainly exists in granulocytes and macrophages which indicates that these two types of cells may have some similar functions, such as in the host defense by mediating efficient microbial killing, as the modulation of vessel tonus, cell-cell interaction, and adhesion, and in response to the presence of damaged low-density lipoproteins.

ETs were first discovered in neutrophils, but it is now accepted that other leukocytes, including monocytes/macrophages, mast cells, basophils, dendritic cells, and eosinophils, can also generate extracellular traps (54). Recently, the presence of ETs has been confirmed in a variety of fish species, including tongue sole, turbot, carp, and Atlantic salmon, and has been attributed to be involved in response to bacterial infection (35, 55–61). Previous studies in carp have revealed that neutrophils release ETs in a time-dependent manner rapidly after stimulation (15 min), which implies that ET formation process is instant (62). In the current study, PMA, *S. aureus, E. coli*, and *E. tarda* induced rapid formation of ETs from flounder HKLs – since 1 hour stimulation was sufficient for the cells to produce ETs. Immunofluorescence staining confirmed that *Po*MPO mainly exists in granulocytes and macrophages, indicating the formation of ETs is mainly associated with neutrophils and macrophages in flounder. And the presence of *Po*MPO within the ET scaffold which provides evidence for extrusion of substances from cytoplasmic granules into the extracellular environment.

ETs have been reported to trap and arrest the proliferation of bacteria rather than kill them (12, 35, 37, 58). In human studies, microorganisms have been shown to be trapped by ETs, and attempts to recover viable entrapped bacteria in the matrix of DNA etc. consequently failed (37). In this study, we were able to enumerate bacterial killing activity after *E. coli* and *S. aureus* exposure – where killing caused by the process of ET formation and probably *Po*MPO activity contributed. However, the killing of *E. tarda* was not present by an unknown reason. We speculate *E. tarda* may evade such mechanisms since they are intracellular bacteria and thus effectively resist the antibacterial effects of ETs (63). In our study, the formation of ETs was always accompanied by a significant increase of ROS and MPO generation, we speculate that a substantial fraction of the MPO in cells is released during ET formation and is potentially active for a long period. In this study, when the ETs MPO were antagonized by anti-r*Po*MPO Abs, the *Po*MPO activity was decreased. Furthermore, in absence of anti-rPoMPO abs, the *E. coli* and *S. aureus* proliferation decreased, which indicated that *Po*MPO contributed to the bacterial killing or bacteriostatic effect.

In conclusion, a *Po*MPO sequence was identified and further characterized which served us to develop antibodies in mice. By use of the antibodies combined with gene expression analysis, we were able to characterize cell types containing *Po*MPO, and we suggest a migration behavior of PoMPO^+^ cells – from the head kidney to blood and peritoneal cavity upon local inflammatory event. We provide evidence for the evolution of MPOs from lower vertebrates to mammals. This work presents new knowledge where *Po*MPO is involved in the antibacterial effect of ETs – as the proliferation of *E. coli* and *S. aureus* was affected. These advances deepen our understanding of the functional role of MPO and “Etosis” in teleost fish.

## Data availability statement

The original contributions presented in the study are included in the article/**Supplementary Material**. Further inquiries can be directed to the corresponding author.

## Ethics statement

The animal study was reviewed and approved by the protocols for animal care and handling were approved by the Animal Care and Use Committee of Ocean University of China (Permit Number: 20180101).

## Author contributions

QG, HC, and RD were associated with the conception of the study and the original draft. QG, XM, and HC performed the experimental and statistical analyses. RD, JX, XT, XS, and WZ edited the manuscript into the final version to be submitted. HC, JX, XT, XS, and WZ provided the funding. All authors contributed to the article and approved the submitted version.
