# Supplementary figures and images for: Characterization of myeloperoxidase and its contribution to antimicrobial effect on extracellular traps in flounder (Paralichthys olivaceus)
Source: Front Immunol. 2023 Jan 26;14:1124813. doi: 10.3389/fimmu.2023.1124813 (PMC9908613; doi:10.3389/fimmu.2023.1124813)

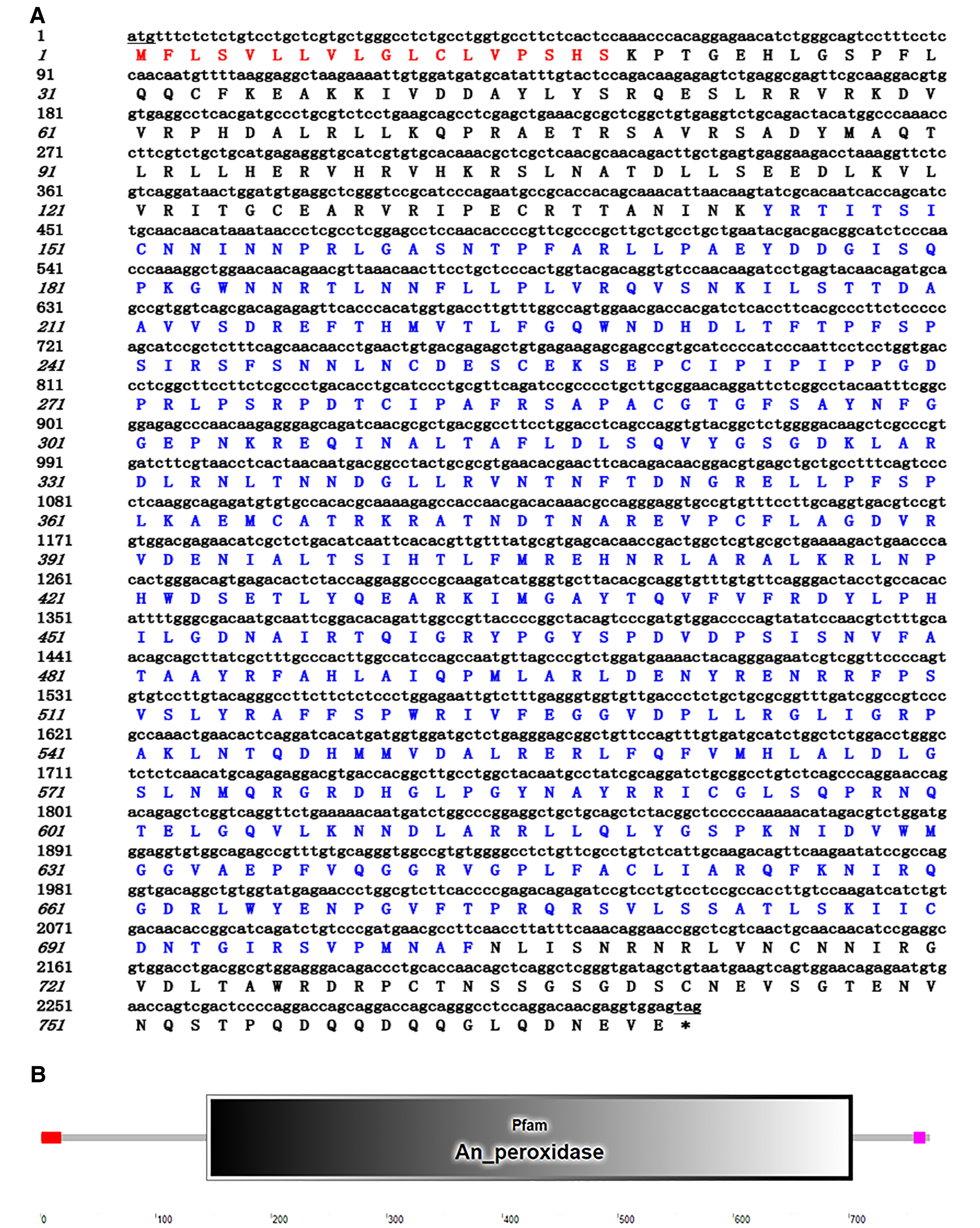

Supplement: Supplementary file 1 [file Image_1.jpeg]

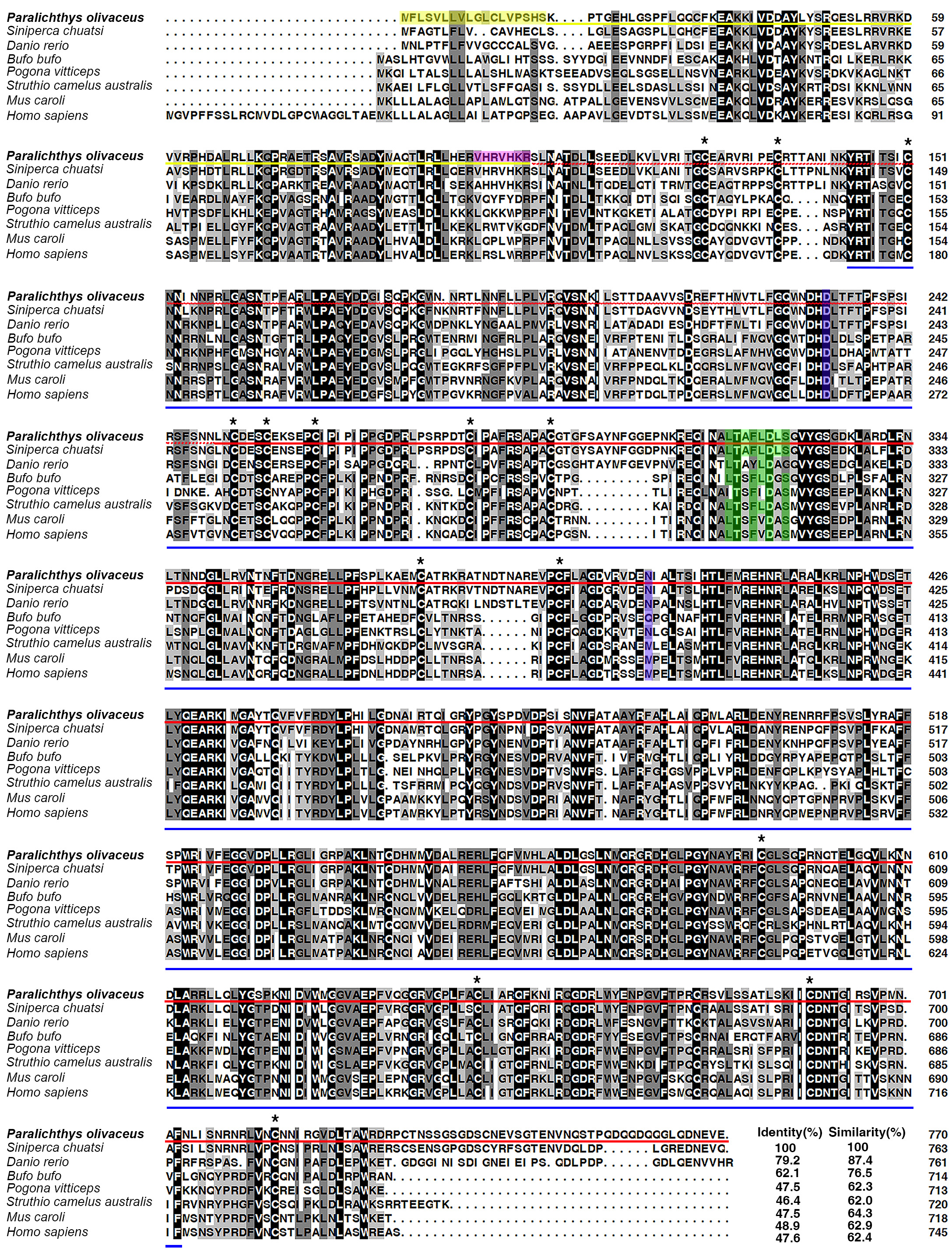

Supplement: Supplementary file 2 [file Image_2.jpeg]

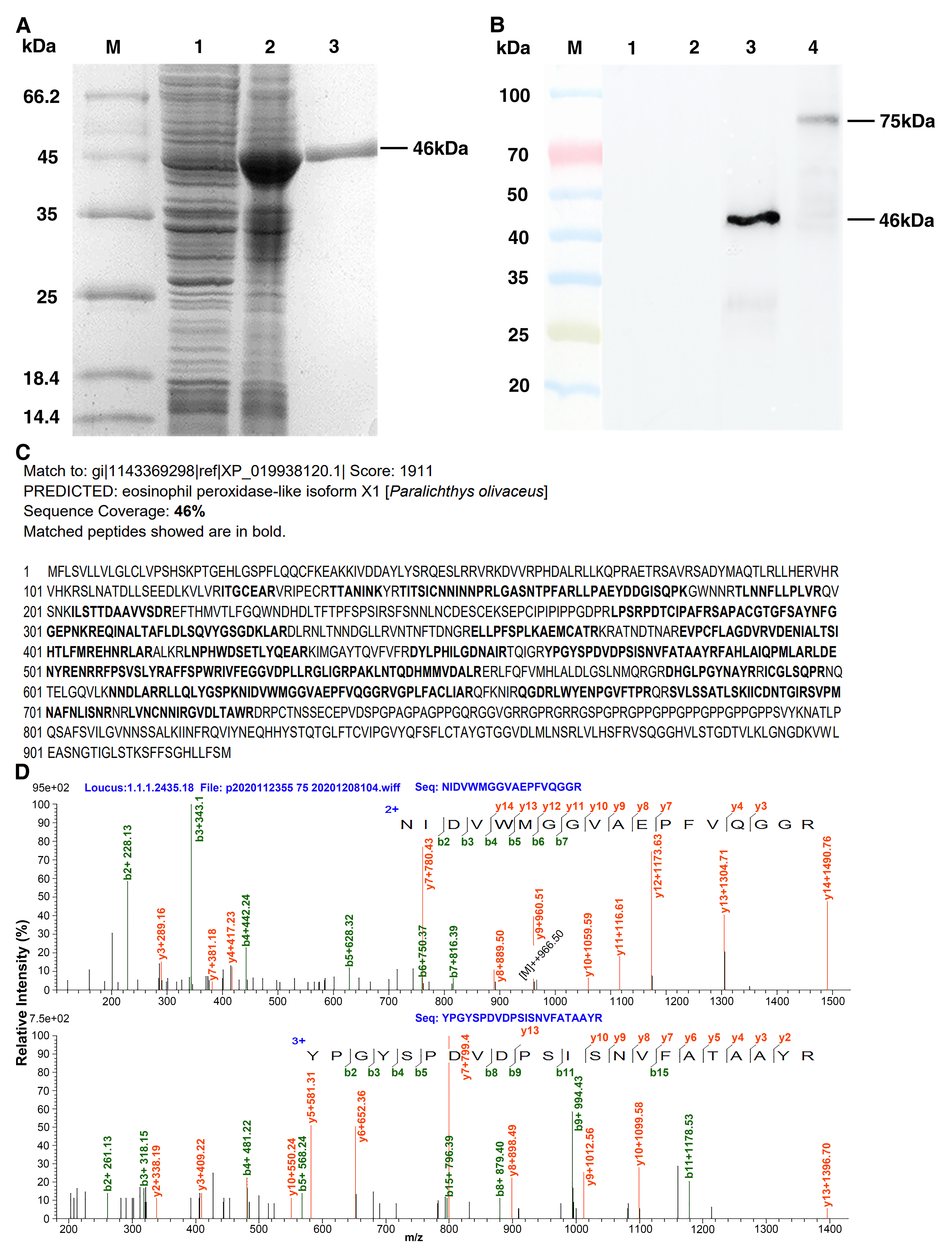

Supplement: Supplementary file 3 [file Image_3.jpeg]

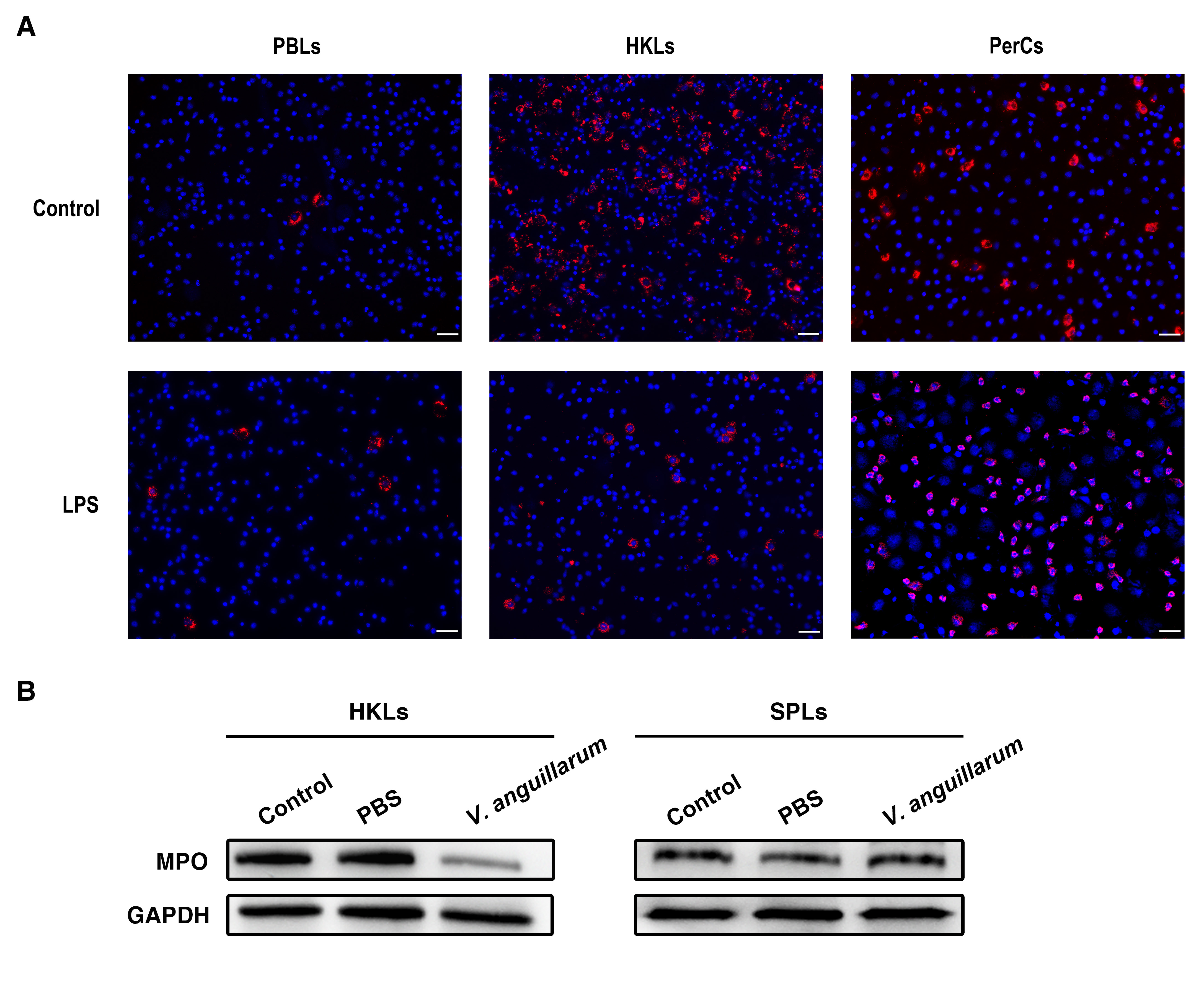

Supplement: Supplementary file 4 [file Image_4.jpeg]

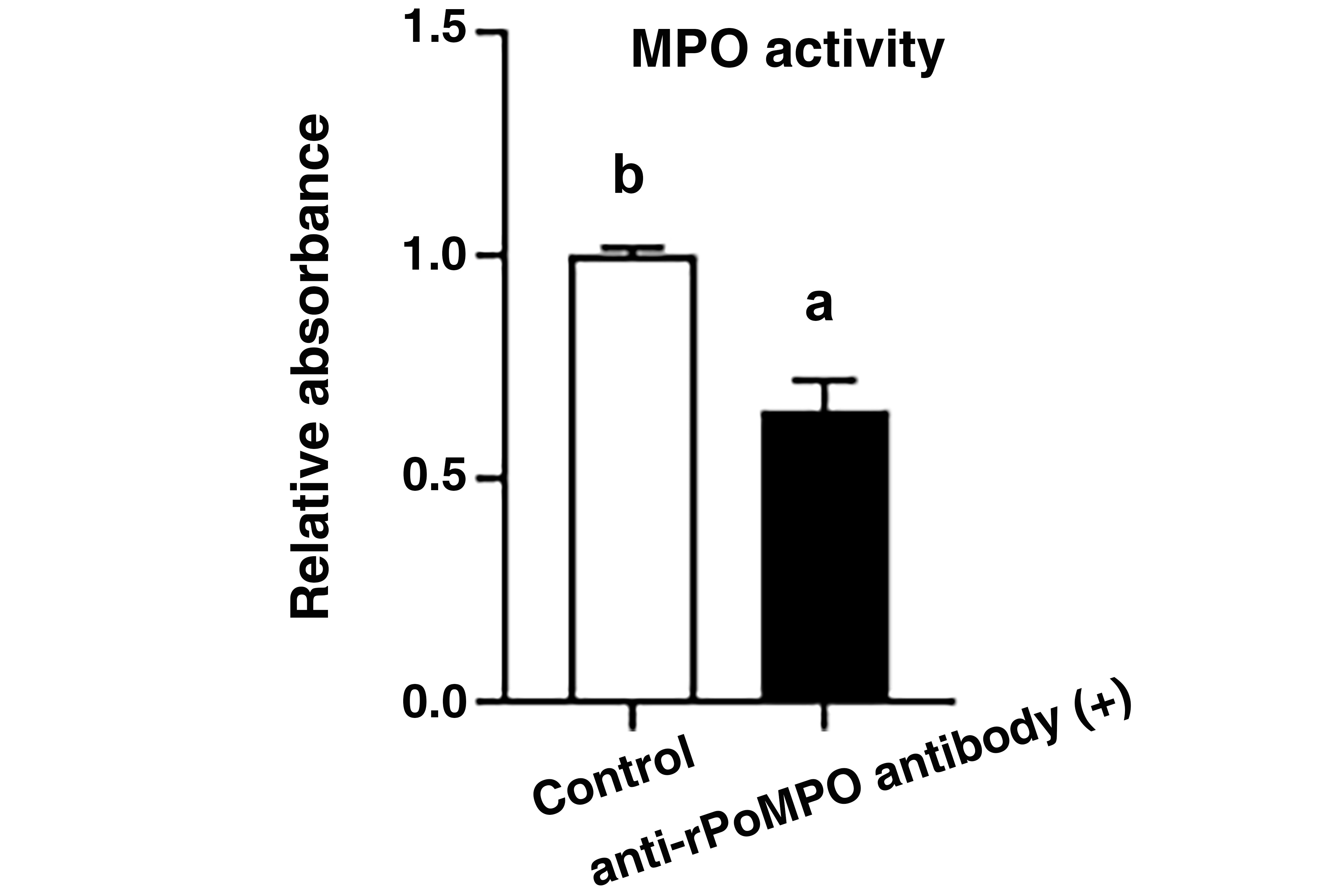

Supplement: Supplementary file 5 [file Image_5.jpeg]
